# Effect on Fœtus

**Published:** 1874-11

**Authors:** 


INFLUENCE OF CHLOROFORM, IN LABOR, UPON THE FŒTUS.—Dr. Zweifel, of the Obstetric Clinics in Strasbourg, has recently made some investigations that would seem to imply that the anæsthetic administered to women in labor has more effect upon the fœtus in utero than is perhaps generally admitted. Dubois has made the statement that anæsthesia of the mother causes rapidity of the fœtal heart-beats. The writer has often observed an appearance of icterus npon newly born children after the use of chloroform, but could not with certainty attribute it to the latter. His attention was first seriously arrested by perceiving in the breath of an infant born a few. hours before, a distinct odor of chloroform. The child had been extracted while the mother was under the influence of anæsthetic, but since the delivery had lain in a room by itself, where no chloroform had been used. Shortly after this, in order to determine positively whether the anæsthetic was conveyed to the fœtus through the maternal circulation, he instituted the following test: a fresh placenta that had just been expelled by a woman to whom chloroform had been administered for only about fifteen minutes, and more than an hour previously, was placed in a close-fitting vessel, having first been cleansed of all adhering clots. The following day when the vessel was opened a decided odor of chloroform was perceived, and further examination proved conclusively the presence of the drug. By still another test, examination of the child's urine, the writer was able to establish the fact of the influence of the anæsthetic upon the fœtus. In conclusion, the writer observes that, since the use of narcotics in general are contra-indicated in infants, it is an important question for obstetricians to decide to just what degree anæsthesia may be carried in women in labor, with impunity to the fœtus.—*Berl. Klin. Woch.*. 21, 1874.

